# The Language of Numbers: Reading Comprehension and Applied Math Problem-Solving

**DOI:** 10.3390/bs15121746

**Published:** 2025-12-17

**Authors:** Dana Sury, Lia Pilchin

**Affiliations:** Faculty of Therapy, Consultation and Educational Support, Beit Berl College, Kfar Saba 44905, Israel; kath.toni89@gmail.com

**Keywords:** reading comprehension, applied math, problem-solving, arithmetic fluency, development

## Abstract

Reading and mathematics are intricately linked through shared cognitive processes that underpin developmental relationships across domains. Despite extensive research on early-grade links between reading and basic arithmetic, gaps persist in understanding how reading comprehension (RC) supports applied math problem-solving (AMP) in older students and non-English contexts. The current study investigates the grade-level relationship between RC and AMP in typically developing Hebrew-speaking fourth (*N* = 41) and eleventh graders (*N* = 43), focusing on the contributions of working memory (WM), reading fluency, and arithmetic fluency. Results indicated significant positive associations between RC and AMP in both age groups. In fourth graders, arithmetic fluency partially statistically mediated the RC-AMP relationship in a cross-sectional mediation model. This indicates that students rely on computational proficiency to translate textual understanding into solutions. In contrast, eleventh graders exhibited a direct RC-AMP link, reflecting advanced comprehension and metacognitive strategies as computational skills are automatized. WM showed stronger correlations with RC and AMP among younger students, whereas these associations were weaker in older students. These findings support a Developmental Linguistic–Cognitive Scaffold Model, highlighting age-related shifts in cognitive and linguistic mechanisms supporting AMP. The results emphasize the need for integrated curricula incorporating RC strategies to enhance mathematical reasoning, particularly in morphologically rich languages like Hebrew.

## 1. Introduction

Reading and mathematics are foundational skills, serving as cornerstones for academic achievement, career success, and mental well-being in modern society (Duncan et al., 2007; Ritchie & Bates, 2013). These domains are intricately linked across development, with early linguistic skills such as phonological awareness, vocabulary, and rapid automatized naming predicting both reading and mathematical proficiency (Little et al., 2017; Moll et al., 2015; Snowling et al., 2021).

Given this interdependence, the present study aims to examine how advanced linguistic skills, specifically reading comprehension (RC), contribute to students’ ability to solve applied math problems (AMP) across two key developmental stages. The current study focuses on fourth and eleventh-graders in a Hebrew-speaking context. It examines how RC interacts with arithmetic fluency, working memory (WM), and reading fluency to support mathematical reasoning.

The maturational relationship between reading and mathematics is further emphasized by their shared reliance on cognitive processes, such as mapping symbolic representations to meaning (Hecht et al., 2001), and the high co-occurrence of difficulties in both domains (Connors et al., 2025; Joyner & Wagner, 2020). These relationships also extend to higher-level skills. For instance, longitudinal studies demonstrate that third-grade RC predicts conceptual understanding and application of mathematical knowledge, suggesting that linguistic abilities are integral to mathematical performance (Grimm, 2008; Little et al., 2022).

Prior research has established links between reading abilities and basic arithmetic skills across childhood (Singer & Strasser, 2017). However, few studies have examined how advanced linguistic skills, such as RC, support complex AMP across critical developmental stages (Grimm, 2008; Little et al., 2022; Vilenius-Tuohimaa et al., 2008). Specifically, studies of middle and high school students, who face increasingly sophisticated mathematical and linguistic demands, are scarce. This study addresses these gaps by examining the RC-AMP relationship in two age groups: fourth graders, who are beginning to engage with complex mathematical concepts, and eleventh graders, who navigate advanced mathematical and linguistic texts in their high school curriculum.

### 1.1. Reading Comprehension in Applied Mathematics

AMP involves verbal descriptions of real-life scenarios requiring the application of learned mathematical operations, necessitating both numerical proficiency and linguistic processing (Hickendorff, 2021). Unlike narrative texts, such as stories or informational articles, mathematical texts have distinct features. They are characterized by density, precision, and hierarchical structure. As a result, they provide fewer contextual cues for unfamiliar terms and increase reliance on specific mathematical vocabulary (Österholm, 2006; Österholm & Bergqvist, 2013; Powell et al., 2020). For instance, terms like “derivative,” “proportion,” or “variable” require precise understanding to solve AMP effectively, and their complexity escalates with grade level, with students expected to master hundreds of mathematical terms throughout their education (Powell et al., 2020; Riccomini et al., 2015). The Language Function Hypothesis (LFH) provides a theoretical framework for this interplay, positing that language serves dual roles in AMP: as a medium for communication and learning (e.g., interpreting word problems) and as a tool for higher-order cognition in mathematical reasoning (e.g., planning solution steps; (Peng et al., 2020).

RC and AMP rely on shared cognitive and linguistic processes, including WM, vocabulary, phonological awareness, and mental schemas, which underpin their developmental relationships (Grimm, 2008; Vilenius-Tuohimaa et al., 2008). WM is a cognitive system that temporarily stores and manipulates information (Cowan, 2008). It is critical for both domains. In AMP, WM supports multi-step operations, numerical processing, and conceptual integration (Friedman et al., 2018; Gaye et al., 2024). RC, WM enables readers to integrate prior knowledge, process syntactic structures, and infer meanings from text (Palladino et al., 2001; Shin, 2020).

Phonological awareness, defined as the ability to manipulate speech sounds (e.g., segmenting “banana” into “ba-na-na”), facilitates decoding in reading (Snowling, 2001; Wagner et al., 1997) and arithmetic fact retrieval, such as recalling multiplication tables (De Smedt & Boets, 2010; Hecht et al., 2001).

Vocabulary, another critical component, supports comprehension by allowing readers to decode and understand complex texts and mathematical terms, reducing cognitive load for higher-order processes like inference-making and problem-solving (Cain & Oakhill, 2014; Riccomini et al., 2015). For example, understanding terms like “ratio” or “exponent” is essential for both RC and AMP tasks.

Beyond individual cognitive processes, instructional approaches are also evident in the contexts of both RC and AMP. For example, schema-based strategies activate prior knowledge through tools such as graphic organizers, KWL (Know-Want-Learned) charts, and semantic mapping (Ogel, 1986). Such strategies enhance both RC (Nassaji, 2007) and AMP (Cook et al., 2020) by fostering a structured understanding of texts and problems. These shared cognitive and linguistic processes underscore the need for integrated approaches to reading and math research and instruction, as they promote comprehension across domains.

### 1.2. The Role of Arithmetic Fluency in Applied Mathematics

Beyond the shared cognitive and linguistic processes linking RC and AMP, arithmetic fluency (i.e., the ability to quickly and accurately perform basic arithmetic operations) plays a critical role in supporting mathematical problem-solving. Arithmetic fluency reduces cognitive load during AMP by enabling automatic retrieval of arithmetic facts, thereby freeing WM resources for higher-order processes such as problem representation, strategy selection, and solution monitoring (Fuchs et al., 2016; Hickendorff, 2021; Kaskens et al., 2022). When computational processes are automatized, students can allocate more cognitive resources to interpreting problem texts, identifying relevant mathematical relationships, and evaluating solution reasonableness.

For younger students who are still developing computational automaticity, arithmetic fluency may serve as a critical bridge between comprehension of problem text and successful mathematical execution. Research demonstrates that arithmetic fluency contributes uniquely to word problem-solving performance (Fuchs et al., 2016; Kaskens et al., 2022). It supports students’ ability to translate linguistic understanding into mathematical computations. During this phase, students may successfully comprehend the linguistic content and structure of a word problem. However, limitations in arithmetic fluency can impede problem-solving by consuming cognitive resources needed to maintain problem information and execute multi-step solutions.

However, as students mature and computational skills become increasingly automatized through practice and instruction, the relative importance of arithmetic fluency may shift. Older students with well-established computational automaticity may rely more directly on RC and conceptual reasoning when solving complex mathematical problems (Csíkos, 2022). This developmental trajectory suggests that the relationship between RC and AMP may be mediated differently across age groups, with arithmetic fluency serving as a more prominent scaffold in earlier grades but diminishing as a mediating mechanism as computational processes become automatic. Understanding these age-related shifts has important implications for curriculum design and instructional sequencing. In particular, they inform when and how to integrate RC strategies into mathematics instruction.

### 1.3. The Effect of Unique Orthography on AMP

In Hebrew, the consistent orthography is characterized by transparent letter-sound mappings and complex morphology with intricate word formation patterns (Share & Daniels, 2016). Such characteristics may enhance RC’s role in AMP by supporting comprehension of dense mathematical texts. For example, Hebrew’s vowel diacritics (nikkud) aid early reading fluency, potentially facilitating precise interpretation of mathematical terms. However, the morphological complexity, such as root-based word structures, may pose challenges for RC in advanced texts, necessitating robust linguistic skills, particularly in dense mathematical texts (Vaknin-Nusbaum, 2025). This language-specific interplay is rarely studied, despite its potential to inform educational practices in Hebrew-speaking contexts and influence how reading fluency and arithmetic fluency support the RC-AMP relationship across development.

### 1.4. The Current Study

The present study integrates theoretical frameworks from both reading and mathematical cognition to understand the mechanisms linking RC to AMP. Building on the Simple View of Reading (Hoover & Gough, 1990), which posits that RC results from the product of decoding and linguistic comprehension, we propose that mathematical word problem-solving similarly requires both domain-general comprehension skills and domain-specific numerical proficiencies. Specifically, we hypothesize that arithmetic fluency serves as a critical mediator between RC and applied math performance. This prediction is grounded in cognitive load theory (Sweller, 2011). When students lack automaticity in arithmetic operations, they must allocate cognitive resources to basic computations, leaving insufficient WM capacity to integrate information from problem texts with mathematical solution procedures (Fuchs et al., 2016; Hickendorff, 2021; Kaskens et al., 2022). Conversely, students with high arithmetic fluency can devote cognitive resources to comprehending problem structure and selecting solution strategies. Furthermore, the current perspective suggests that the mediating role of arithmetic fluency differs between elementary and secondary students. In elementary grades, where arithmetic operations constitute a substantial portion of word problem demands, fluency is expected to strongly mediate the RC–AMP relationship. However, as students progress to secondary mathematics involving more complex operations (algebra, proportional reasoning), arithmetic fluency may become necessary but insufficient, with RC exerting a stronger direct influence on problem-solving success. This perspective aligns with models showing that component skills become integrated and automated with increased proficiency (Geary, 2011).

Despite extensive research on the reading-mathematics relationship, significant gaps persist in understanding how RC supports AMP across different ages, particularly in typically developing populations and non-English linguistic contexts. Most studies focus on early grades (Fuchs et al., 2022; Hickendorff, 2021) or atypical populations (Connors et al., 2025; Friedman et al., 2018), with limited exploration of older students facing complex mathematical texts (Fuchs et al., 2015; Österholm, 2006). Additionally, research in transparent orthographies like Finnish may not generalize to morphologically complex orthographies like Hebrew, where RC develops more slowly due to root-based word structures and gradual removal of vowel diacritics (Share & Daniels, 2016).

The present study makes three distinct contributions addressing these gaps. First, while longitudinal studies like Grimm (2008) have demonstrated that early RC predicts later mathematical achievement, they provide snapshots at discrete time points rather than examining how cognitive mechanisms linking RC to AMP differ qualitatively across development. Our cross-sectional comparison of fourth and eleventh-graders tests whether the same cognitive pathways operate at both ages or whether fundamentally different mechanisms emerge. Second, whereas prior work in transparent orthographies has documented strong reading-math connections (Vilenius-Tuohimaa et al., 2008) Hebrew’s unique combination of consonantal script, optional vowel diacritics, and root-based morphology creates linguistic demands that may amplify RC’s role in mathematical reasoning, particularly for older students encountering abstract mathematical vocabulary. Third, by testing arithmetic fluency as a mediator and examining how its role changes with age, we address a critical gap in understanding mechanisms through which linguistic skills support mathematical problem-solving across schooling.

Accordingly, the current study investigates the RC-AMP relationship in typically developing Hebrew-speaking fourth and eleventh-grade students, examining the roles of arithmetic fluency, WM, and reading fluency. Fourth graders, transitioning from basic arithmetic to complex concepts like fractions, and eleventh graders, navigating advanced algebra and geometry, provide a perspective on how these cognitive and linguistic factors interact to support AMP across critical educational transitions. We address three research questions: (1) What is the association between AMP and RC in fourth and eleventh-grade students? (2) How do WM, reading fluency, and arithmetic fluency contribute to this relationship, and does arithmetic fluency mediate the RC-AMP association? (3) Do these associations differ between fourth and eleventh graders?

Based on the theoretical framework, the current study will test three hypotheses.

(1) RC will be significantly associated with AMP in both grades, reflecting linguistic comprehension’s fundamental role in interpreting mathematical word problems. (2) Arithmetic fluency will show a mediating pattern in the relationship between RC and AMP in fourth grade, as predicted by cognitive load theory: when basic arithmetic is less automatized, limited WM will constrain students’ ability to translate comprehension into successful solutions; and (3) In 11th grade, the mediating role of arithmetic fluency will be reduced or absent. This shift occurs because automatized arithmetic operations no longer serve as a primary bottleneck, enabling RC to directly shape complex mathematical reasoning.

## 2. Materials and Methods

### 2.1. Participants

The current study included 46 fourth graders and 43 eleventh-graders, Hebrew-speaking participants. However, 5 participants from the fourth-grade group were excluded from the study due to standard scores below −2.0 in the arithmetic and reading (i.e., single word decoding) fluency tests. Thus, data from 41 fourth graders and 43 eleventh graders were analyzed in the present study. The fourth-grade group included 18 females and 23 males with a mean age of 9 years and 7 months (*SD* = 4 months). The eleventh-grade group included 14 females and 29 males with a mean age of 16 years and 7 months (*SD* = 5 months), all enrolled in advanced-level mathematics within the Israeli matriculation system. According to the Israeli mathematics curriculum (Ministry of Education, 2018), in fourth grade, the curriculum introduces complex concepts beyond whole numbers (e.g., fractions), where basic skills like arithmetic fact retrieval become tools for applying conceptual knowledge in AMP. In eleventh grade, students prepare for high school final mathematics exams, requiring logical thinking expressed through quantitative, visual, and verbal methods (Hilu & Schori-Eyal, 2024).

Post hoc power analyses were conducted using G*Power 3.1.9.7 (Faul et al., 2009) to evaluate sample adequacy for detecting the observed effects (see Section 3.2 for detailed results).

### 2.2. Procedure

Testing took place in participants’ homeroom classrooms or a quiet adjacent room at their school. Participants completed five assessments measuring WM, arithmetic fluency, AMP solving, RC, and reading fluency. The AMP and RC tests were administered in a group setting, with the examiner providing instructions before distributing test sheets. Participants then completed the tasks independently (see Section 2.3). The arithmetic fluency, reading fluency, and WM tests were conducted individually in a quiet room to ensure privacy and minimize distractions.

### 2.3. Materials

Participants completed the following standardized assessments:

1. Working Memory: The Backward Digit Span subtest from the Wechsler Intelligence Scale for Children (Wechsler, 2003) assessed WM, measuring the ability to hold and manipulate information in short-term memory. The test has a reliability of 0.93. It consists of six items, each with six trials. Participants repeat number sequences in reverse order (e.g., for “5-6,” the correct response is “6-5”). Both trials of each item are administered, regardless of Trial 1 accuracy. Each correct trial scores 1 point. The total raw score is the sum of items scored.
2. Arithmetic fluency: The Math Fluency subtest of the Woodcock-Johnson IV Test of Achievement (WJ IV ACH; Schrank et al., 2014). This standardized subtest measures participants’ ability to solve simple addition, subtraction, and multiplication facts quickly and accurately. The same measure was administered to both 4th-grade and 11th-grade students, as automatic retrieval of basic arithmetic facts remains foundational for mathematical problem-solving across development, even as students engage with increasingly complex operations.

Students were given a three-minute time limit to complete as many arithmetic problems as possible from a series of progressively more difficult items (160 items). Raw scores represent the total number of correct responses provided within the time limit. The Math Fluency subtest demonstrates strong psychometric properties, with a reliability of 0.90 (Schrank et al., 2014).

3. Applied Math Problem-Solving (AMP): The Applied Problem-Solving subtest from the KeyMath-3 Diagnostic Assessment (Connolly, 2007) evaluated participants’ ability to interpret and solve contextual math problems. The test has a reliability of 0.89 and covers topics from algebra, geometry, measurement, and data analysis, requiring both computational skills and conceptual knowledge. Participants were allowed to use draft paper and calculators and were instructed to guess or skip items they found challenging. The test was translated into Hebrew by the research team. The raw score is the number of correct answers.
4. Reading Comprehension: RC was assessed using age-appropriate informative texts followed by multiple-choice questions evaluating literal, interpretive, and evaluative comprehension.

Fourth graders completed an RC test based on the expository text *The Magic of Dragonflies* (Katzir et al., 2017). This informative text, approximately 200 words in length and comprising six short passages about dragonflies, was followed by 13 multiple-choice questions (max scoring 32, range of scores: 10–32). The test assesses understanding of passage content, inference of word meanings from context, syntactic relationships conveyed through conjunctions and prepositions, and logical connections across text sections, skills essential for comprehending academic texts. The raw scores represent the total points for all test items, based on one point per section. The exam consists of 13 questions, each containing a varying number of sections. The reliability of this measure is 0.71 (Katzir et al., 2017).

Eleventh graders completed an RC test drawn from a previous administration of the Psychometric Entrance Test by the Israeli National Institute for Testing and Evaluation. Because age-appropriate standardized RC measures are not available for secondary students in Hebrew, we selected a test that assesses the same comprehension skills required for academic success: understanding of passage content, inference of word meanings from context, syntactic relationships (e.g., via conjunctions and prepositions), and logical connections across text sections. The test consisted of one page-length expository text (approximately 600 words across five passages) describing the history and symbolism of the Israeli flag, followed by 13 comprehension questions (range of scores 9–13). To ensure validity, participating schools confirmed that students had not previously encountered these specific test materials. Raw scores were calculated as the number of correct answers. Although this measure has not been subjected to independent validation studies, the Psychometric Entrance Test is a high-stakes standardized assessment used for university admissions in Israel. It was developed with rigorous psychometric procedures by the National Institute for Testing and Evaluation (n.d.).

5. Reading Fluency: The single-word reading subtest from the “Alef-Ad-Taf” standardized Hebrew reading diagnosis assessment (Shany et al., 2006) measured reading speed and accuracy. Participants read a list of 36 context-free words aloud as quickly and accurately as possible. Scores included reading speed (time in seconds) and reading accuracy (number of errors).

### 2.4. Statistical Analysis

To examine relationships between RC, arithmetic fluency, AMP, WM, and reading fluency, Spearman correlation tests were conducted using raw scores from the AMP, WM, fluency tests, and the percentage of correct answers from the RC tests. Note that the RC test was the only test that differed between age groups due to the inability to match the text and level of questions between these two age groups.

To identify the strongest predictors of AMP, linear regression analyses were performed separately for each age group, with AMP as the dependent variable and WM, arithmetic fluency, and RC as independent variables.

### 2.5. Ethics

The study was approved by the Israeli Chief Scientist’s Office of the Ministry of Education and the Ethics Committee of Beit-Berl College. Written informed consent was obtained from all participants and their parents. Participants were informed of their right to withdraw from the study at any time without consequence.

## 3. Results

### 3.1. Descriptive Statistics

Descriptive statistics, including means and standard deviations, were calculated for the variables assessed in the present study: AMP, RC, arithmetic fluency, WM, and reading fluency (speed and accuracy). These statistics are presented separately for fourth-grade (*N* = 41) and eleventh-grade (*N* = 43) participants in Table 1. As anticipated, performance differed across age groups, with eleventh graders generally exhibiting higher mean scores.

Shapiro–Wilk tests indicated significant deviations from normality for all variables (all *p* < 0.001), suggesting skewness that may influence subsequent correlation and regression analyses. These non-parametric distributions support the use of non-parametric or robust methods, such as Spearman’s rank-order correlations and bootstrapping, in the analyses that follow.

### 3.2. Statistical Power Analysis

Post hoc power analyses were conducted using G*Power 3.1.9.7 (Faul et al., 2009) to evaluate the adequacy of the sample sizes for detecting the observed effects, with α = 0.05 (two-tailed) for all analyses.

For the correlation between RC and applied math problem-solving, achieved power was 0.73 for fourth-grade (*N* = 41, r = 0.38) and 0.96 for eleventh grade (*N* = 43, *r* = 0.50).

For the multiple regression models with three predictors (reading comprehension, arithmetic fluency, and WM), achieved power was 0.94 for fourth-grade (*N* = 41, *R*^2^ = 0.31, *f*^2^ = 0.445) and 0.99 for eleventh grade (*N* = 43, *R*^2^ = 0.45, *f*^2^ = 0.805).

For the critical mediation a-path (RC—arithmetic fluency), achieved power was 0.88 (*N =* 41, *r* = 0.45).

All analyses met or approached the conventional power threshold of 0.80 (Cohen, 1988), except for the fourth-grade correlation (0.73), which remains acceptable for detecting medium-sized effects (Lakens, 2022). These power estimates demonstrate that the sample sizes were adequate to detect the medium-to-large effects observed in this study.

### 3.3. Associations Between Reading, Arithmetic, WM, and AMP

To examine associations between AMP and cognitive-linguistic variables (RC, arithmetic fluency, WM, and reading fluency), Spearman’s rank-order correlations were computed separately for each age group (see Table 2 and Table 3). Due to significant non-normality (Shapiro–Wilk tests, all *p* < 0.001), bootstrap resampling (1000 samples) was used to estimate 95% confidence intervals (CIs) for the correlation coefficients, enhancing precision given the modest sample sizes.

In both age groups, AMP was significantly positively correlated with RC and arithmetic fluency. Across both groups, WM was significantly correlated with arithmetic fluency, supporting a robust link between WM and computational skills. Reading fluency, speed, and accuracy were significantly correlated in both groups, indicating that faster reading was associated with fewer errors.

Age-related differences emerged in this analysis: in fourth graders, RC was significantly correlated with arithmetic fluency, WM, and reading speed, whereas these associations were non-significant in eleventh graders. Conversely, WM was positively and significantly correlated with AMP in the fourth-grade group. In the eleventh-grade group, WM was negatively correlated with reading fluency speed and accuracy, indicating that higher WM capacity is associated with better reading fluency. This pattern was not observed in the fourth-grade group. Reading speed was also significantly correlated with AMP in the eleventh-grade group and with RC in the fourth-grade group.

Fisher’s r-to-z transformations revealed no significant difference in the strength of the correlation between AMP and RC across age groups (*z* = −0.63, *p* = 0.52), nor between AMP and arithmetic fluency (*z* = −1.26, *p* = 0.21).

### 3.4. Regression Analyses for Predicting AMP Performance by Age Group

Separate multiple regression analyses were conducted for each age group to identify predictors of AMP. Predictor variables included RC, WM, and arithmetic fluency. Bootstrapping procedures (1000 samples) were used to estimate robust 95% CIs for regression coefficients (see Table 4).

For fourth graders, the model was statistically significant [*F* (3, 37) = 5.48, *p* = 0.003], explaining 30.8% of the variance in AMP scores (*R*^2^ = 0.308, adjusted *R*^2^ = 0.251). Arithmetic fluency was a significant positive predictor, while RC and WM were not significant. Collinearity diagnostics indicated acceptable levels of multicollinearity.

For eleventh graders, the model was also significant, *F* (3, 39) = 10.48, *p* < 0.001, explaining 44.6% of the variance (*R*^2^ = 0.446, adjusted *R*^2^ = 0.404). RC was the strongest predictor, followed by arithmetic fluency. WM was not a significant predictor. Tolerance and VIF values indicated no multicollinearity concerns.

Variable selection for regression and mediation models was guided by both theoretical considerations and preliminary correlational analyses. Reading fluency (speed and accuracy) was excluded based on the theoretical prediction that reading fluency influences AMP primarily through its contribution to RC development rather than having a direct independent effect.

Post-hoc hierarchical regression analysis for eleventh grade confirmed this prediction: adding reading fluency speed to the model (which included reading comprehension, arithmetic fluency, and WM) did not significantly improve prediction [Δ*R*^2^ = 0.006, *F* (1, 38) = 0.42, *p* = 0.52].

WM was retained despite non-significant regression coefficients because of: (a) significant bivariate correlations with AMP (*r* = 0.36, *p* < 0.05), RC (*r* = 0.37, *p* < 0.05), and arithmetic fluency (*r* = 0.52, *p* < 0.001) in fourth-grade, (b) very strong correlations with arithmetic fluency in both grades (*r* = 0.52–0.63, *p* < 0.001), and (c) theoretical importance as a domain-general cognitive capacity. The developmental shift in WM’s relationship with AMP from significant bivariate correlation in Grade 4 to non-significant in Grade 11 warranted investigation in multivariate models for both grades.

### 3.5. Mediation Analysis

To examine whether arithmetic fluency mediates the relationship between RC and AMP, separate mediation analyses were conducted for each age group using Jamovi. 2.7.5 (R Core Team, 2025; The jamovi project, 2025). Given the significant non-normality observed in the data, bootstrapping procedures with 5000 resamples were applied to estimate robust 95% confidence intervals (CIs) for indirect, direct, and total effects (see Table 5).

In the fourth-grade group, the indirect effect of RC on AMP through arithmetic fluency was statistically significant, accounting for 59.3% of the total effect. The direct effect was non-significant, while the total effect was significant, indicating partial mediation. Path coefficients revealed a significant effect of RC on arithmetic fluency and of arithmetic fluency on AMP. Thus, results revealed a significant indirect effect in the fourth-grade group, consistent with partial mediation.

In the eleventh-grade group, the indirect effect was not statistically significant, accounting for 13.8% of the total effect. The direct effect remained significant, suggesting no mediation in this age group.

The significant indirect effect in fourth-grade (*p* = 0.01, bootstrapped *CI* [0.01, 0.05]) demonstrates that the sample size was adequate to detect the theoretically predicted mediation. Post hoc power analysis for the a-path (RC—Arithmetic Fluency) yielded power = 0.88, well above the conventional 0.80 threshold. The non-significant indirect effect in eleventh grade (*p* = 0.23, bootstrapped *CI* [−0.01, 0.04]) is consistent with the theoretical expectation that arithmetic fluency plays a diminished mediating role as students develop automaticity in basic mathematical operations and rely more directly on RC for solving complex mathematical problems.

## 4. Discussion

The present study explored the developmental relationship between RC and AMP in typically developing Hebrew-speaking fourth and eleventh-grade students. It also examined how WM, reading fluency, and arithmetic fluency contribute to this connection. The findings reveal a robust RC-AMP association across both age groups, with notable shifts between grades in the role of arithmetic fluency and the contributions of WM and reading fluency. These results offer empirical support for the Language Function Hypothesis (Peng et al., 2020), and the Structure Correspondence Hypothesis (Cui et al., 2024), which posit that linguistic and cognitive processes jointly scaffold mathematical reasoning.

### 4.1. RC-AMP Relationship Across Development

The significant positive correlations between RC and AMP in both fourth graders and eleventh graders affirm the hypothesis that RC is a key predictor of AMP performance. This is consistent with prior research (Grimm, 2008; Little et al., 2022), which highlights the role of linguistic comprehension in mathematical cognition. Importantly, this association persists across grades, suggesting that RC remains a foundational skill even as students transition from basic arithmetic to more abstract mathematical reasoning.

In fourth graders, RC’s effect on AMP was partially mediated by arithmetic fluency. This indicates that younger students rely on computational proficiency to operationalize their understanding of mathematical texts. In contrast, eleventh graders demonstrated a direct RC-AMP link, with no significant mediation by arithmetic fluency. This shift suggests that older students can leverage higher-order comprehension and metacognitive strategies to interpret mathematical texts, as their computational skills are more automatized (Csíkos, 2022).

Notably, the AMP tasks used in this study required minimal reading: short texts with illustrations and limited linguistic complexity. Yet, RC remained a significant predictor of performance. This emphasizes RC’s role not only in decoding and interpreting text but also in constructing mental schemas that integrate textual, numerical, and visual information. Solving AMP requires students to identify relevant details, filter extraneous information, and map problem structures to mathematical operations (Cook et al., 2020; Jitendra et al., 2002). These processes mirror those involved in text understanding, where readers build coherent mental representations by integrating new information with prior knowledge.

Hebrew’s orthographic features may further amplify RC’s role in AMP. Its morphological complexity, including root-based word structures and infrequent use of vowel diacritics (nikkud), increases the linguistic demands of mathematical texts. For older students, who are less accustomed to fully vocalized texts, interpreting mathematical vocabulary may require more deliberate lexical processing (Vaknin-Nusbaum, 2025; Weiss et al., 2015). These findings highlight the need for explicit instruction in mathematical vocabulary and text comprehension, particularly in Hebrew-speaking contexts.

A concrete example illustrates how Hebrew’s morphological complexity affects AMP: Consider the mathematical term Ratio (in Hebrew, pronounced as yachas, meaning ‘ratio’ or ‘proportion’). Without vowel diacritics, this three-letter root (Ya-cha-S) could be read multiple ways depending on the vowel pattern. The same root also forms related words: *hiityachasut* (meaning ‘relation’ or ‘reference’), *mityaches* (meaning ‘relates to’), and *yichus* (meaning ‘attribution’). When students encounter this term in a word problem, for example, “Find the ratio between the numbers” they must: (1) Accurately decode the unvocalized word Ratio (yachas), (2) Distinguish it from morphologically related terms with different meanings, (3) Retrieve the precise mathematical meaning (‘ratio’) rather than general meanings (‘relation’) and, (4) Integrate this understanding with numerical information to solve the problem.

For younger students still learning to navigate unvocalized text, Step 1 consumes cognitive resources. For older students with developed decoding skills, Steps 2–3 remain challenging when mathematical vocabulary is not explicitly taught, as the morphological system creates semantic ambiguity that general Hebrew proficiency does not resolve. Hence, RC plays such a critical role in Hebrew mathematical problem-solving: the linguistic demands extend beyond simple word recognition to precise lexical disambiguation and domain-specific meaning activation. Thus, explicit mathematical vocabulary instruction, emphasizing both morphological structure and domain-specific meanings, is particularly important in Hebrew-speaking contexts.

Taken together, the results affirm the dual nature of AMP as both a numerical and linguistic task. RC supports students’ ability to construct coherent mental representations, interpret problem requirements, and select appropriate operations. This link evolves with age: younger students rely on arithmetic fluency to bridge RC and AMP, while older students demonstrate a direct link, reflecting more advanced comprehension and strategic processing.

### 4.2. Contributions of Arithmetic Fluency, WM, and Reading Fluency

#### 4.2.1. Arithmetic Fluency

Beyond the overall RC-AMP relationship, individual cognitive factors contributed differentially across age groups. Arithmetic fluency significantly predicted AMP performance in both age groups, consistent with research showing that automaticity reduces cognitive load and frees resources for problem-solving (Baker & Cuevas, 2018; Hickendorff, 2021). In fourth graders, arithmetic fluency mediated the RC-AMP association, suggesting that computational proficiency serves as a bridge between linguistic comprehension and mathematical execution. This aligns with developmental models of cognitive resource allocation, which posit that younger students rely more heavily on basic skills to support higher-order reasoning (Z. Lin, 2024).

In eleventh graders, arithmetic fluency remained a significant predictor of AMP but did not mediate the RC-AMP link. This suggests an age-related shift toward direct reliance on RC and conceptual reasoning. As students mature, their computational skills become more automatized, allowing them to focus on interpreting complex mathematical texts and applying abstract concepts. These findings support the integration of RC strategies into math instruction, particularly in early grades, to scaffold students’ ability to interpret and solve word problems.

Moreover, the results underscore the importance of arithmetic fluency not only as a foundational skill but also as a stepping stone toward more sophisticated mathematical reasoning. Instructional practices that combine computational fluency with linguistic comprehension, such as schema-based instruction and vocabulary integration, may enhance students’ ability to navigate text-rich mathematical environments.

#### 4.2.2. Working Memory

WM was significantly correlated with arithmetic fluency in both age groups, supporting its role in basic computational skills (Balhinez & Shaul, 2019; Berg, 2008). However, WM was only linked to AMP and RC in fourth graders and did not emerge as a significant predictor in regression models. This diverges from prior studies that emphasize WM’s role in both RC and AMP across development (Fuchs et al., 2022; Ji & Guo, 2023).

Several factors may explain this discrepancy. First, the AMP task’s design, including the use of calculators and visual aids, may have reduced WM demands by providing external supports. Second, stronger WM capacity in older students may have enabled compensation, diminishing its direct impact on performance. Third, the Backward Digit Span task may not fully capture the WM processes relevant to AMP, such as updating, inhibition, and integration. Indeed, recent meta-analyses suggest that tasks tapping the phonological loop have weaker associations with AMP than those assessing complex span or executive functioning (Ji & Guo, 2023).

Interestingly, RC was associated with WM in younger students but not in older ones. This aligns with research showing that WM supports RC by enabling readers to hold and manipulate information, process syntactic structures, and integrate meaning across sentences (Palladino et al., 2001; Shin, 2020). However, as students’ decoding skills become more automatized and their comprehension strategies more refined, reliance on WM may diminish (Orsolini et al., 2023; Seigneuric & Ehrlich, 2005).

These findings underscore the importance of task-sensitive WM measures and suggest that phonological WM may be less relevant for advanced mathematical reasoning. Future studies should incorporate broader WM assessments, including complex span and executive functioning tasks, to better capture its role in RC and AMP.

#### 4.2.3. Reading Fluency

Reading fluency showed differences in its association with RC and AMP between grades. In fourth graders, RC was significantly correlated with reading speed, suggesting that rapid decoding supports comprehension. In eleventh graders, RC was linked to reading accuracy, reflecting a shift toward precise word recognition and strategic processing. This pattern aligns with developmental trends, indicating a transition from fluency-based support to more refined comprehension strategies in adolescence (Orsolini et al., 2023; Peng et al., 2018).

Reading speed was significantly correlated with AMP only in eleventh graders, suggesting that decoding efficiency supports performance in more complex tasks. However, the reading fluency task used fully vocalized Hebrew words, which older students rarely encounter. While the vowel diacritics aid phonological clarity, their infrequent use may hinder fluency and comprehension in adolescents (Share & Daniels, 2016; Weiss et al., 2015). Thus, accuracy may become a more sensitive indicator of fluency in older readers.

These findings highlight the need to consider orthographic features when assessing reading fluency and its role in RC and AMP. In Hebrew, morphological complexity and script familiarity interact with grade level changes in decoding and comprehension, shaping how students engage with mathematical texts.

### 4.3. Developmental Linguistic–Cognitive Scaffold Model

**Figure 1:**
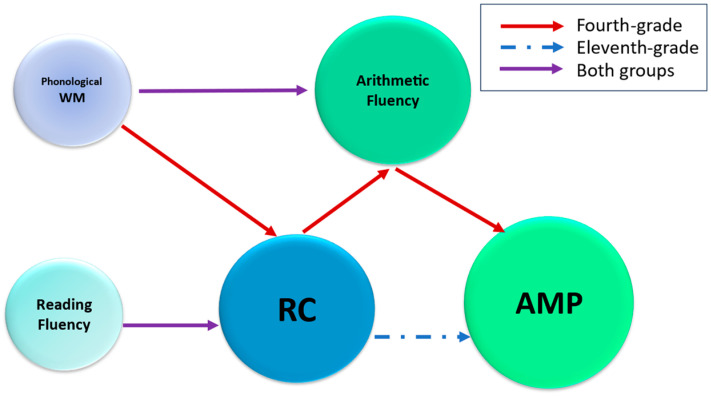
Developmental Linguistic–Cognitive Scaffold Model (DLCSM) for RC and AMP. The model depicts the evolving relationship between RC and AMP in Hebrew-speaking fourth and eleventh-graders. In fourth graders, RC influences AMP indirectly via arithmetic fluency (red arrows), reflecting dependence on computational proficiency. In eleventh graders, a direct RC-AMP link emerges (dashed arrow), driven by advanced comprehension and metacognitive strategies. WM and reading fluency serve as dynamic supports, with phonological WM’s influence decreasing and reading fluency shifting from speed-based to accuracy-driven with age. Hebrew’s morphological complexity and vowel diacritics (nikkud) modulate these interactions, highlighting linguistic demands in AMP.

Based on current findings, we propose a Developmental Linguistic–Cognitive Scaffold Model (DLCSM, see Figure 1) to explain the evolving association between RC and AMP across age groups. In this model, RC consistently predicts AMP performance, but the mechanisms shift across development. For younger students, RC supports AMP indirectly through arithmetic fluency, reflecting reliance on computational proficiency to operationalize textual understanding. In contrast, older students demonstrate a direct RC–AMP link, suggesting greater reliance on higher-order comprehension and metacognitive strategies. WM and reading fluency serve as dynamic support that change with development: phonological WM contributes to RC in younger students but diminishes in influence as comprehension strategies mature, while reading fluency transitions from speed-based decoding to accuracy-driven lexical processing. Hebrew’s orthographic features, particularly morphological complexity and optional vowel diacritics, modulate these relationships across development.

The DLCSM integrates cognitive resource theory (Sweller, 2011) with developmental models of skill acquisition (Anderson, 1982). According to cognitive load theory, WM capacity is limited, and tasks requiring multiple concurrent processes compete for finite cognitive resources. For younger students, a lack of arithmetic automaticity forces allocation of WM to basic computations, constraining resources available for text integration and strategic problem-solving. Arithmetic fluency thus serves as a bottleneck: even students with strong RC cannot fully leverage their linguistic skills if cognitive resources are consumed by effortful calculation. As computational skills become automatized through practice and development, they transition from controlled to automatic processing (Anderson, 1982), freeing cognitive resources for direct application of RC to mathematical reasoning. This progression explains why arithmetic fluency mediates the RC-AMP association in fourth graders but not in eleventh graders, whose automatized computation no longer constitutes a resource bottleneck.

The theoretical novelty of DLCSM lies in integrating three dimensions that prior frameworks treat separately. First, whereas the Language Function Hypothesis (Peng et al., 2020) and Structure Correspondence Hypothesis (Cui et al., 2024) describe language-mathematics relationships as relatively stable across development, DLCSM proposes developmental reorganization wherein pathways linking linguistic and mathematical skills fundamentally change as component skills become automatized. The shift from indirect (mediated by arithmetic fluency) to direct RC-AMP relationships reflects qualitative change in cognitive architecture, not merely quantitative differences. Second, DLCSM specifies arithmetic fluency as the key mediating mechanism in early development, providing mechanistic detail absent from broader frameworks. This specificity enables testable predictions about when and why language-math connections should be stronger or weaker. Third, DLCSM incorporates orthographic and morphological features as moderators, predicting that RC’s influence on AMP varies systematically across writing systems. For Hebrew specifically, morphological complexity amplifies RC’s importance, particularly for older students navigating unvocalized mathematical vocabulary.

These theoretical distinctions have practical implications. For elementary students, where arithmetic fluency mediates the RC-AMP relationship, instruction should explicitly integrate RC strategies, arithmetic fluency practice, and word problem-solving as an interconnected system. For secondary students, where direct RC-AMP links emerge, instructional emphasis should shift toward advanced comprehension strategies for mathematical texts, metacognitive monitoring, and explicit connections between comprehension and conceptual reasoning. In Hebrew-speaking contexts, morphological complexity necessitates explicit mathematical vocabulary instruction addressing root-based word structures, a principle applicable to any language where orthographic features create additional linguistic demands. For assessment, separately evaluating reading comprehension, arithmetic fluency, and applied problem-solving enables identification of whether poor AMP performance reflects linguistic, computational, or integration difficulties, allowing targeted interventions.

### 4.4. Limitations

Several limitations should be considered when interpreting these findings. First, the sample was drawn from Hebrew-speaking students in two schools in northern Israel. While the sample sizes are consistent with prior research and provided adequate power (0.73–0.99) to detect medium-to-large effects typical of RC and AMP associations, they limit the ability to detect smaller effects and reduce generalizability to the broader Hebrew-speaking population. The socio-economic, cultural, and geographic diversity within Israel is not fully represented, and generalizability to other Hebrew-speaking populations, educational contexts, or linguistic systems requires empirical verification. Future research would benefit from larger, stratified samples across diverse educational settings to establish the robustness of these findings and uncover more nuanced relationships.

Second, the orthographic properties of Hebrew, particularly its vowel representation and morphological complexity, may influence the specific cognitive pathways observed. Although these linguistic features may shape developmental trajectories, the consistency of our findings with international research (Fuchs et al., 2016; Peng et al., 2020) suggests that the shift from mediated to direct relationships between reading and mathematics may reflect universal cognitive processes.

Third, the cross-sectional design limits causal inferences about the age-related change of RC-AMP relationships. Although our findings align with a developmental model in which arithmetic fluency mediation diminishes with age, longitudinal studies are needed to examine within-individual changes over time and to rule out cohort effects or other alternative explanations for the observed grade differences. Future research should track these relationships into high school, where mathematical reasoning becomes more complex.

Finally, methodological considerations warrant attention. The use of different RC tests across age groups, though age-appropriate, may introduce variability. Additionally, the Backward Digit Span task may not fully capture the WM demands inherent in AMP. Incorporating broader cognitive measures, such as complex span tasks and executive function assessments, could better elucidate WM’s role in these associations. Taken together, these limitations underscore the importance of interpreting the findings within the context of previous studies and working hypotheses, as well as pointing to important directions for future research.

### 4.5. Future Direction

The current findings open several important avenues for future research, addressing both methodological refinement and theoretical extensions. An important methodological challenge highlighted by this study concerns the assessment of RC in secondary students. Standardized RC tests with established norms for older students are scarce. This gap forced us to adapt materials from university entrance examinations. While arithmetic fluency can be adequately assessed using existing standardized instruments such as the Woodcock-Johnson (appropriate across age ranges and targeting the specific skill of automatic arithmetic retrieval), RC assessment in secondary populations requires further development.

Future research would benefit from instruments specifically designed to assess the RC processes most relevant to AMP. Such measures should isolate the linguistic and cognitive skills involved in understanding mathematical texts, including comprehension of technical vocabulary, syntactic structures common in AMP, and its logical relationships, while minimizing the confounding influence of prior mathematical knowledge itself. Such assessment tools should also isolate domain-specific reading strategies such as identifying relevant versus irrelevant information, recognizing problem schemas (e.g., “compare” problems vs. “change” problems), and monitoring comprehension of quantitative relationships, which may explain variance beyond general comprehension ability. The development of such measures would enable more precise investigation of how RC supports mathematical problem-solving and may provide a diagnostic tool for identifying students whose mathematical difficulties stem from reading challenges rather than mathematical reasoning deficits.

Beyond foundational arithmetic fluency, future research might also examine more complex numerical competencies (e.g., fluency with fractions, negative numbers, algebraic manipulation) or conceptual mathematical knowledge as potential mediators or moderators of the reading-mathematics relationship (Xu et al., 2022). Indeed, the current study identified arithmetic fluency as an important mediator in the RC-AMP association for elementary students. However, a comprehensive understanding of this link requires investigation of additional cognitive mechanisms and contextual factors that may mediate or moderate the observed effects. From the existing evidence, mathematical vocabulary emerges as one of the central factors that could serve as a mediator in the RC-AMP relationships (i.e., words or phrases that express mathematical concepts or procedures; (Lariviere et al., 2025; X. Lin et al., 2021). Beyond general reading comprehension, students must understand specialized mathematical terminology, symbolic notation, and linguistic structures common in word problems (e.g., comparative statements, conditional logic). Mathematical vocabulary may independently mediate the association between RC and AMP, particularly as problems increase in complexity. Mathematical vocabulary may also convey prior mathematical content knowledge, such as domain-specific knowledge in areas of proportional reasoning, algebraic thinking, or geometric concepts, as the relationship between RC and AMP may depend on students’ knowledge of the mathematical content domains being assessed.

The cross-sectional design of the current study provides a valuable perspective of the RC-AMP association at two distinct ages, but cannot address questions of developmental change within individuals or causal mechanisms. Thus, future studies that will employ Longitudinal designs may reveal how the relationships among reading comprehension, arithmetic fluency, and applied math evolve within individuals. Such designs could identify critical transition points where the cognitive architecture of problem-solving reorganizes, determine whether early arithmetic fluency predicts later problem-solving success even after controlling for concurrent fluency, and test whether the developmental trajectory differs for students with varying initial skill profiles.

## 5. Conclusions

Recent international assessments, including TIMSS 2023 (Mullis et al., 2020) and PISA (OECD, 2023), have revealed a concerning global decline in student achievement in mathematics and science. These trends underscore the urgent need to reexamine the foundational skills that support mathematical reasoning and the application of mathematical knowledge in real-world contexts.

The present study highlights applied AMP as a dual-domain task, requiring both numerical proficiency and linguistic comprehension. Students must construct mental representations that integrate textual and quantitative information, making RC a critical component of mathematical reasoning.

This interdependence between reading and mathematics calls for integrated curriculum designs that explicitly address the linguistic demands of mathematical problem-solving. Incorporating RC strategies, such as schema-based instruction, into mathematics education may enhance students’ ability to interpret and navigate complex mathematical texts, particularly in morphologically rich languages like Hebrew.

## Figures and Tables

**Table 1:** Means and Standard Deviations of Raw Scores for Participants’ Performance Across Administered Tests.

Test	Fourth-Grade (*N* = 41)	Eleventh-Grade (*N* = 43)
AMP	14.37 (1.99)	32.81 (1.40)
RC (% correct)	72.02 (20.00)	92.66 (8.12)
Arithmetic Fluency	74.76 (15.85)	128.14 (17.47)
WM	12.92 (3.37)	24.27 (3.01)
Single Word Reading: Speed (in seconds)	71.95 (33.84)	26.74 (3.67)
Single Word Reading: Accuracy (number of errors)	5.17 (2.66)	2.28 (1.68)

**Table 2:** Spearman Correlations and 95% Confidence Intervals Among Variables in the Fourth-Grade Group (*N* = 41).

Variable	1	2	3	4	5
1. AMP	-				
2. RC	0.38 ** (0.13, 0.64)	-			
3. Arithmetic Fluency	0.58 *** (0.27, 0.72)	0.62 *** (0.33, 0.75)	-		
4. WM	0.36 * (−0.01, 0.56)	0.37 * (0.14, 0.65)	0.52 *** (0.42, 0.79)	-	
5. Reading Fluency- Speed	0.11 (−0.28, 0.33)	−0.38 * (−0.63, −0.11)	−0.26 (−0.48, 0.11)	−0.18 (−0.46, 0.13)	-
6. Reading Fluency- Accuracy	0.17 (−0.18, 0.43)	−0.16 (−0.42, 0.18)	−0.10 (−0.54, 0.03)	0.11 (−0.27, 0.34)	0.56 *** (0.15, 0.66)

**Note**. Spearman ρ values are reported with 95% confidence intervals (in parentheses) based on 1000 bootstrap samples. df = 39 for all comparisons. * p < 0.05, ** p < 0.01, *** p < 0.001.

**Table 3:** Spearman Correlations and 95% Confidence Intervals Among Variables in the eleventh-Grade Group (*N* = 43).

Variable	1	2	3	4	5
1. AMP	-				
2. RC	0.50 *** (0.33, 0.74)	-			
3. Arithmetic Fluency	0.36 * (0.19, 0.67)	0.20 (−0.08, 0.49)	-		
4. WM	0.22 (0.000, 0.55)	0.20 (−0.08, 0.49)	0.63 *** (0.40, 0.78)	-	
5. Reading Fluency- Speed	−0.33 * (−0.49, 0.09)	−0.25 (−0.38, 0.21)	−0.34 * (−0.50, 0.07)	−0.49 *** (−0.67, −0.18)	
6. Reading Fluency- Accuracy	−0.21 (−0.48, 0.10)	−0.46 ** (−0.64, −0.13)	−0.21 (−0.44, 0.15)	−0.43 ** (−0.64, −0.14)	0.46 ** (0.27, 0.72)

**Note**. Spearman ρ values are reported with 95% confidence intervals (in parentheses) based on 1000 bootstrap samples. df = 39 for all comparisons. * p < 0.05, ** p < 0.01, *** p < 0.001.

**Table 4:** Regression Coefficients and Collinearity Statistics for the Fourth and Eleventh Grade Groups.

Predictor	B	SE	β	t	*p*	95% CI (Bootstrapped)	Tolerance	VIF
**Fourth-grade**								
RC	0.02	0.02	0.18	1.07	0.29	[−0.027,0.055]	0.66	1.51
Arithmetic fluency	0.06	0.03	0.49	2.46	0.02	[0.015, 0.130]	0.48	2.10
WM	−0.05	0.11	−0.09	−0.50	0.62	[−0.55, 0.13]	0.58	1.72
**Eleventh-grade**								
RC	0.08	0.02	0.50	4.04	0.00	[0.032, 0.128]	0.10	1.07
Arithmetic fluency	0.03	0.01	0.39	2.50	0.02	[0.007, 0.056]	0.60	1.67
WM	−0.02	0.07	−0.05	−0.34	0.74	[−0.171, 0.129]	0.60	1.67

**Note.** *CI = Confidence Interval. Bootstrapped 95% confidence intervals based on 1000 samples. All predictors entered simultaneously.*

**Table 5:** Mediation Analysis Results for RC, Arithmetic Fluency, and AMP by Age Group.

Effect	Estimate	SE	95% CI [Lower, Upper]	Z	*p*	% Mediation
**Fourth-Grade (*N* = 41)**						
Indirect (RC → Arithmetic Fluency → AMP)	0.02	0.01	[0.01, 0.05]	2.41	0.01	59.3
Direct (RC → AMP)	0.06	0.02	[−0.02, 0.05]	0.88	0.38	40.7
Total	0.04	0.01	[0.01, 0.07]	2.67	0.01	100
**Path Coefficients**						
RC → Arithmetic Fluency	0.45	0.09	-	4.80	<0.001	-
Arithmetic Fluency → AMP	0.05	0.02	-	2.28	0.02	-
RC → AMP	0.02	0.02	-	0.88	0.38	-
**Eleventh Grade (*N* = 43)**						
Indirect (RC → Arithmetic Fluency → AMP)	0.01	0.01	[−0.01, 0.04]	1.2	0.23	13.8
Direct (RC → AMP)	0.08	0.02	[0.04, 0.12]	3.5	<0.001	86.2
Total	0.09	0.03	[0.04, 0.14]	3.58	<0.001	100
**Path Coefficients**						
RC → Arithmetic Fluency	0.45	0.32	-	1.4	0.16	-
Arithmetic Fluency → AMP	0.03	0.01	-	2.91	0.004	-
RC → AMP	0.08	0.02	-	3.5	<0.001	-

*Note. CI* = Confidence Interval. Mediation percentages reflect the proportion of the total effect accounted for by the indirect pathway (RC → Arithmetic Fluency → AMP). Estimates are based on standardized coefficients. Confidence intervals were derived using bootstrapping procedures with 5000 resamples. *p* < 0.05 is considered statistically significant.

## Data Availability

The data presented in this study are available upon reasonable request from the corresponding author.

## Abbreviations

The following abbreviations are used in this manuscript:

AMP	Applied Math Problem
DLCSM	Developmental Linguistic–Cognitive Scaffold Model
LFH	Language Function Hypothesis
RC	Reading Comprehension
WM	Working Memory
